# The effectiveness of psychological support interventions for those exposed to mass infectious disease outbreaks: a systematic review

**DOI:** 10.1186/s12888-021-03602-7

**Published:** 2021-11-24

**Authors:** Alison Doherty, Valerio Benedetto, Catherine Harris, Paul Boland, Danielle L. Christian, James Hill, Gita Bhutani, Andrew J. Clegg

**Affiliations:** 1 Synthesis, Economic Evaluations and Decision Science (SEEDS) Group, Faculty of Health & Care, University of Central Lancashire (UCLan), Preston, PR1 2HE UK; 2 Lancashire & South Cumbria NHS Foundation Trust & University of Liverpool, Liverpool, UK

**Keywords:** Review, Pandemics, Public health, Mental health, Interventions, Mass outbreaks

## Abstract

**Background:**

Mass outbreaks such as pandemics are associated with mental health problems requiring effective psychological interventions. Although several forms of psychological interventions may be advocated or used, some may lack strong evidence of efficacy and some may not have been evaluated in mass infectious disease outbreaks. This paper reports a systematic review of published studies (PROSPERO CRD:42020182094. Registered: 24.04.2020) examining the types and effectiveness of psychological support interventions for the general population and healthcare workers exposed to mass infectious disease outbreaks.

**Methods:**

A systematic review was conducted. Randomised Controlled Trials (RCT) were identified through searches of electronic databases: Medline (Ovid), Embase (Ovid), PsycINFO (EBSCO) and the Cochrane Library Database from inception to 06.05.2021 using an agreed search strategy. Studies were included if they assessed the effectiveness of interventions providing psychological support to the general population and / or healthcare workers exposed to mass infectious disease outbreaks. Studies were excluded if they focused on man-made or natural disasters or if they included armed forces, police, fire-fighters or coastguards.

**Results:**

Twenty-two RCTs were included after screening. Various psychological interventions have been used: therapist-guided therapy (*n* = 1); online counselling (*n* = 1); ‘Emotional Freedom Techniques’ (*n* = 1); mobile phone apps (*n* = 2); brief crisis intervention (*n* = 1); psychological-behavioural intervention (*n* = 1); Cognitive Behavioural Therapy (*n* = 3); progressive muscle relaxation (*n* = 2); emotional-based directed drawing (*n* = 1); psycho-educational debriefing (*n* = 1); guided imagery (*n* = 1); Eye Movement Desensitization and Reprocessing (EMDR) (*n* = 1); expressive writing (*n* = 2); tailored intervention for patients with a chronic medical conditions (*n* = 1); community health workers (*n* = 1); self-guided psychological intervention (*n* = 1), and a digital behaviour change intervention (*n* = 1). Meta-analyses showed that psychological interventions had a statistically significant benefit in managing depression (Standardised Mean Difference [SMD]: -0.40; 95% Confidence Interval [CI]: − 0.76 to − 0.03), and anxiety (SMD: -0.72; 95% CI: − 1.03 to − 0.40). The effect on stress was equivocal (SMD: 0.16; 95% CI: − 0.19 to 0.51). The heterogeneity of studies, studies’ high risk of bias, and the lack of available evidence means uncertainty remains.

**Conclusions:**

Further RCTs and intervention studies involving representative study populations are needed to inform the development of targeted and tailored psychological interventions for those exposed to mass infectious disease outbreaks.

**Supplementary Information:**

The online version contains supplementary material available at 10.1186/s12888-021-03602-7.

## Background

Over a decade before the outbreak of the COVID-19 pandemic in 2020, healthcare organisations across the world were preparing for an influenza pandemic of unpredictable scale and impact [1–5], involving increased rates of morbidity and mortality among the general population, high healthcare demands, and considerable psychological stress amongst healthcare workers [1, 2, 4]. It is evident that the effects of the COVID-19 pandemic are pervasive affecting the mental health of many of those exposed [6, 7], including healthcare workers [8–11].

The effects on healthcare workers is a concern, given their importance in preventing and managing the consequences of pandemics [12, 13]. A mass outbreak puts healthcare workers in unprecedented situations including dilemmas over how to balance their own physical and mental healthcare needs along with those of their patients [13]. Experience with the SARS outbreak in 2003 highlighted how the acute stress of an outbreak can impact on the mental health and wellbeing of healthcare workers and how this, in turn, can affect their ability to care for patients [14, 15]. During the SARS outbreak many healthcare workers reduced their working hours and face-to-face involvement with patients [14, 15]. Two years after the mass infectious disease outbreak, healthcare practitioners that had treated SARS patients had elevated signs of chronic stress compared to healthcare practitioners not treating SARS patients [15]. SARS-CoV-2 is a virus that can cause COVID-19, a mass infectious disease. Reducing the mental health impact of those exposed to such mass infectious disease outbreaks is fundamental to the continued provision of health and social care [2, 5]. However, the planning and delivery of such psychological support may differ within and between countries [16–18].

Although several forms of psychological interventions [19] may be advocated or used, some are recognised as being harmful, others lack strong evidence of efficacy and some have not been evaluated in mass infectious disease outbreaks [16, 19–22]. Importantly, specific population groups such as children and young people, ethnic minorities, and people on low incomes, may be more vulnerable to mental health problems associated with mass infectious disease outbreaks and require targeted interventions [23–28]. Such uncertainties call for the development and implementation of effective targeted interventions for all those exposed to mass infectious disease outbreaks [11, 16, 26]. Despite several systematic reviews assessing interventions to manage psychological problems associated with different types of mass outbreaks [28–31], doubts remain due to certain shortcomings. Some focus on different types of disasters (not just epidemics or pandemics), on interventions for children only, for healthcare workers only, and/or exclude recent evidence [28–31]. Consequently, we conducted a systematic review to identify the types of psychological interventions used in previous mass infectious disease outbreaks (similar to COVID-19) and during the COVID-19 pandemic to support the general population and healthcare workers, and how effective these interventions have been. Findings are expected to provide evidence-based information to inform research, policy and practice.

## Methods

### Search strategy

Our systematic review was conducted according to a pre-registered protocol (PROSPERO 2020 CRD:42020182094. Registered: 24.04.2020), following established PRISMA guidance and reporting standards [32, 33]. We identified studies through searches of electronic databases, including Medline (Ovid), Embase (Ovid), PsycINFO (EBSCO) and the Cochrane Library Database, using a predetermined search strategy and pre-piloted screening tool (Additional files 1 & 2). Databases were searched from inception to 06.05.2021.

### Study selection

#### Inclusion

We included Randomised Controlled Trials (RCTs) including Cluster RCTs and Parallel RCTs that assessed the comparative effectiveness of interventions providing psychological support to the general population (all ages) and/or healthcare workers (e.g. nurses, doctors) exposed to mass infectious disease outbreaks including COVID-19, H1N1, swine flu, SARS, Ebola, and MERS. Psychological support could include interventions such as cognitive behavioural, psycho-social or psycho-educational interventions. Any comparator was included, for example: comparison with no intervention, with usual care, comparisons between a psychological intervention and another type of psychological intervention(s), or pharmacological intervention(s). Effectiveness was assessed using any measure of changes in psychological or mental health impact: specifically reduced depression, anxiety or stress levels measured by a recognised outcome measurement tool such as the Patient Health Questionnaire depression scale (PHQ-9) or the Generalized Anxiety Disorder scale (GAD-7) or the Depression, Anxiety and Stress Scale (DASS-21). Relevant systematic reviews were only used to identify any RCTs that may not have already been identified by the review’s initial electronic searches.

#### Exclusion

Non-RCT studies were excluded. Studies were excluded if they involved armed forces, police, firefighters, coastguards; terrorism / war; or, man-made or natural disasters (e.g. tsunamis). Abstracts, editorials, commentaries, or opinion pieces were excluded, as were studies where the full text was not available or if they were not published in English. Table 1 summarises the eligibility criteria for the review. Titles and abstracts of papers from the searches were screened independently by pairs of reviewers (AJD/VB/CH/AJC), using an eligibility criteria screening tool (Additional file 2). Full-text manuscripts of studies that met the criteria at the title and abstract screening stage were retrieved and screened independently by the pairs of reviewers (AJD/VB/CH/AJC) using the same criteria.

**Table 1:** Eligibility criteria

Population	Intervention	Comparator	Outcomes	Study Design
Healthcare staff exposed to mass infectious disease outbreaks e.g. nurses, physicians, allied health professionals, healthcare support staff. General population, (all ages) including children, adolescents, adults, patients exposed to mass infectious disease outbreaks. Any healthcare setting or any community setting, in any country.	Psychological or psychosocial interventions used to support the mental health of those exposed to mass infectious disease outbreaks, including: H1N1 (a type of influenza A virus), swine flu, Severe Acute Respiratory Syndrome (SARS - *SARS-CoV-2 is a virus that can cause COVID-19, a disease*), Ebola, Middle East Respiratory Syndrome (MERS), and COVID-19.	Any comparator. For example, comparison with no care, with usual care, with another type of psychological support intervention, or with a pharmacological intervention.	Measurable changes (or perceived levels of changes) in mental health disorders including depression, anxiety or stress.	Randomised Controlled Trials (RCTs). Cluster RCTs.

### Data extraction and quality assessment

The pairs of two reviewers (AJD/VB/CH/AJC) independently extracted each study’s data using a pre-piloted data extraction form, checking each other’s extraction. Data were extracted into the following categories: study (first author, year); country; setting; study aims; mass outbreak (type); participant characteristics; intervention(s); comparator(s); and outcomes. The pairs of reviewers independently assessed the quality of included studies using the Cochrane Risk of Bias (RoBv2) Tool [34] and checked one another’s assessments. Any discrepancies at any stage were resolved through discussions to reach mutual consensus.

### Data analysis

Studies were synthesised narratively with tabulation of results. Where studies presented continuous outcome measures of depression, anxiety and stress, they were pooled through meta-analysis presenting results as point estimates and 95% confidence intervals (CI). Although outcomes were measured on different scales, they were based on the same underlying construct, allowing standardised weighted mean differences (SMDs) to be estimated. Given the variation in the studies, random-effects models were used to pool outcomes. Heterogeneity was assessed through visual inspection of forest plots and the calculation of the I^2^ statistics. Pre-planned sub-group analyses explored the influence of study setting, participants and risk of bias. Meta-analyses were conducted using Review Manager version 5.4.1 (Cochrane Collaboration 2020).

## Results

**Fig. 1:**
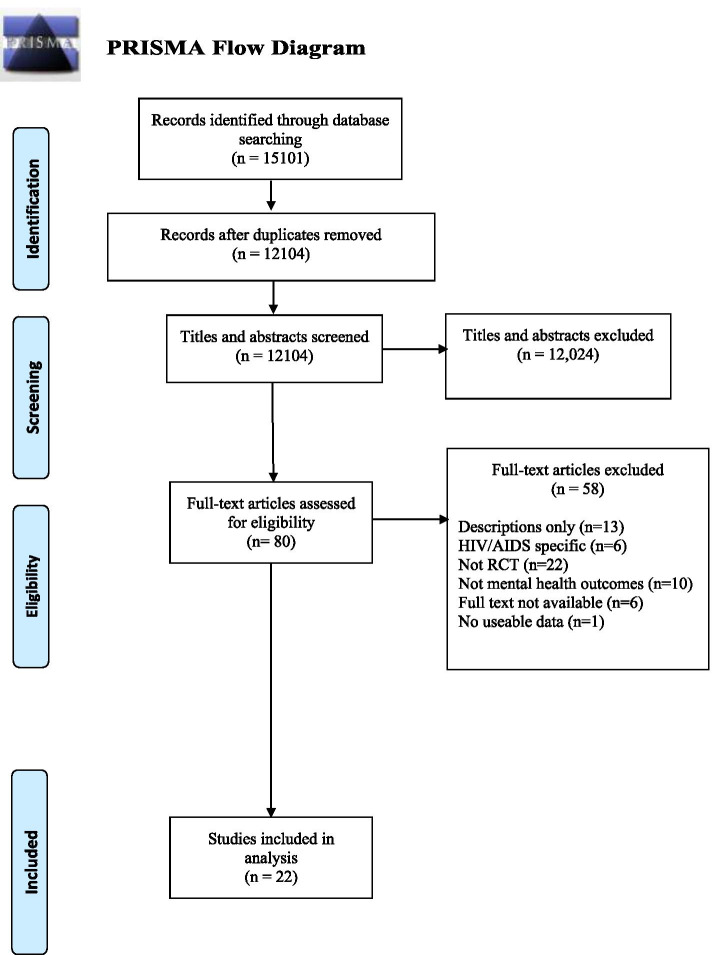
PRISMA Flowchart

A combined total of 12,104 citations were identified from the database searches after removal of duplicates. No further eligible RCTs were identified from other sources (reference checks of relevant reviews). Twenty-two papers met the eligibility criteria and reported information for quality appraisal and data extraction [35–56]. Fig. 1 summarises the study selection process. Table 2 provides a summary of the included studies.

**Table 2:** Summary of included studies

First author, year, (Study type)	Country; setting	Population	Intervention and follow-up	Comparator	Outcome
Al-Alawi, 2021 (RCT) [35]	Oman; Community.	Adults in the general population living in Oman during COVID-19 pandemic (*N* = 60) but data only available for *N* = 46 (*n* = 22 intervention group; *n* = 24 control group); of which: 78% female; mean age 28.51 years (Standard Deviation (SD): 8.70 years).	Internet-based (email-delivered) therapist-guided online therapy focusing on symptoms of anxiety and depression. One session per week for 6 weeks. Follow-up: 6 weeks.	Control group received an automatic weekly newsletter via email containing self-help information and tips to cope with psychological distress associated with COVID-19.	**Outcome measures used**: Patient Health Questionnaire-9 (PHQ-9) and General Anxiety Disorder-7 (GAD-7) scale. Analysis of covariance indicated a significant reduction in the GAD-7 scores (F1,43 = 7.307; *P* = .01) between the two groups after adjusting for baseline scores. GAD-7 scores of participants in the intervention group were considerably more reduced than those of participants in the control group (β = − 3.27; *P* = .01). A greater reduction in mean PHQ-9 scores was observed among participants in the intervention group (F1,43 = 8.298; *P* = .006) than those in the control group (β = − 4.311; *P* = .006). Although the levels of anxiety and depression reduced in both study groups, the reduction was higher in the intervention group (*P* = .049) than in the control group (*P* = .02).
Carbone, 2021 (RCT) [36]	Italy; Community.	Adults in the general population living in Italy during the COVID-19 pandemic (*N* = 54) (*n* = 26 intervention group; *n* = 28 control group): 77% female (*N* = 41); mean age: 34.34 years (SD: 8.27 years).	Online counselling session focusing on reducing clinical symptoms and increasing wellbeing during the first COVID-19 Italian lockdown. One session (only) lasting 60 min. Follow-up: none.	Control group were on a waiting list.	**Outcome measures used:** Self-report questionnaires: BSI Inventory; Positive and Negative Affect Schedule (PANAS); State-Trait Inventory of Cognitive and Somatic Anxiety (STICSA); The Warwick-Edinburgh Mental Wellbeing Scale (WEMWBS) Compared to the control group, the intervention group showed a significant reduction in anxiety (M ± SD 36.65 ± 8.35 vs 48.04 ± 11.51; *p* < 0.001).
Dincer, 2021 (RCT) [37]	Turkey; Hospital.	Hospital-based nurses (*N* = 72 completed the intervention) (*n* = 35 intervention group; *n* = 37 control group): 88.8% female; mean age: 33.45 years (SD: 9.63 years).	Brief online form of Emotional Freedom Techniques (EFT) aimed at prevention of stress and anxiety in nurses involved in treatment of COVID-19 patients. One session (only) lasting 20 min. Follow-up: none.	Control group received no intervention.	**Outcome measures used**: Subjective Units of Distress (SUD) scale, State-Trait Anxiety Inventory (STAI-1), burnout scale via online survey • The mean anxiety score reduction on the post-test for the intervention group was highly significant (*p* < 0.001). • The mean post-test anxiety score for the control group was not statistically significantly different.
Fiol-DeRoque, 2021 (Parallel RCT) [38]	Spain; Hospital and community.	Healthcare workers (*N* = 482) (*n* = 248 intervention group; *n* = 234 control group): 83.2% female; median age: 42 years; 33.4% nurses; 31.7% physicians;30.5% nurse assistants; 81.3% worked in a hospital setting; 12.7% worked in primary care; 6% worked in home-care settings.	A psychoeducational, mindfulness-based mHealth intervention entitled PsyCovidApp. Two-week duration. Follow-up: by telephone between 24 h and 10 days post-intervention using the same questionnaire used at baseline.	Control app comprising brief written information about the mental healthcare of healthcare workers during the COVID-19 pandemic (*adapted from a set of materials developed by the Spanish Society of Psychiatry*).	**Outcome measures used:** A composite of depression, anxiety, and stress: Depression Anxiety and Stress Scale (DASS21). • The Intervention Group presented significantly lower overall scores (suggesting improved mental health) at 2 weeks than the Control Group (adjusted standardized mean difference − 0.29; 95% CI: − 0.48 to − 0.09; *p* = 0.004). • The Intervention significantly improved symptoms of: • Anxiety (*adjusted standardised mean differences*) (− 0.26; 95% CI-0.45 to − 0.08; *p* = .004); • Stress (*adjusted standardised mean differences*) (− 0.30; 95% CI − 0.50 to − 0.09; *p* = .003); • PTSD (*adjusted standardised mean differences*) (− 0.20; 95% CI-0.37 to − 0.03; *p* = 0.01) • No significant differences were observed for symptoms of depression.
Gharaati Sotoudeh, 2020 (RCT) [39]	Iran; Hospital	Patients with diagnosis of COVID-19 (*N* = 30) (*n* = 16 intervention group; *n* = 14 control group): 53.3% female; age range: 20 to 70 years; mean age Intervention Group: 41.92 years (SD: 12.2 years); mean age Control Group: 44.7 years (SD: 14.2 years).	Brief Crisis Intervention Package. Duration: four 60-min sessions for 1 month. Weekly sessions comprised: (1) greetings and introduction to the package; (2) adjustments skills; (3) responsibility and factualism; (4) spirituality. Follow-up: 1 month.	Routine care.	**Outcome measures used**: DASS21, symptom checklist (SCL-25), Quality of Life Assessment developed by the World Health Organisation (WHO-QOL) • The t-test results showed that the average score of depression, anxiety and stress after the intervention was statistically significant compared to the pre-test (*p* < 0.05). • The results of the ANCOVA showed statistically significant differences as a result of the intervention vs usual care in depression, anxiety, stress, mental health, and quality of life (*p* < 0.05).
Kong, 2020 (RCT) [40]	China; Hospital.	Patients with COVID-19 (*N* = 144) (*n* = 13 intervention group; *n* = 13 control group): 51.4% female; 51.4% aged over 50 years.	Psychological-Behavioural Intervention which included breathing exercises and psychosocial support. Duration: 10 days. Breathing exercises performed daily for 20 mins in the morning. Psychosocial support lasted approx. 15 mins. Delivered by two trained medical staff. Follow-up: after 10 days treatment.	Usual care.	**Outcome measures used:** Hospital Anxiety and Depression Scales for Anxiety and Depression (HADS-A and HADS-D); Perceived Social Support Scale (PSSS) self-report. After a 10-day intervention, the Hospital Anxiety and Depression Scale-Anxiety (HADS-A) score (Mean 6.15 +/− 3.579) and the Hospital Anxiety and Depression Scale-Depression (HADS-D) score (5.92 +/− 3.730) were significantly reduced in the Intervention Group (both *p* < 0.001).
Li, 2020 (RCT) [41]	China; Hospital.	Patients with COVID-19 diagnosis (*N* = 93) (*n* = 47 intervention group; *n* = 46 control group): 64.5% female; mean age: 48 years; 20.4% had chronic diseases.	Cognitive Behavioural Therapy (CBT). Comprised: cognitive intervention, relaxation techniques training, problem-solving training, and social support strategy. Performed once a day in the morning, taking 30 mins to complete. Recorded by nurses. Delivered face-to-face and adjusted to suit individual patient’s needs. Follow-up: not reported.	Routine care.	**Outcome measure used:** Chinese version of the Depression, Anxiety and Stress Scale (DASS-21) • A significant decrease in the means for scales of depression, anxiety and total DASS-21 (*DASS-12 is the Chinese version of the Depression Anxiety and Stress Scale-21*) were found in both intervention (*p* < 0.001) and control groups (*p* = 0.001). Participants in the Intervention Group had a bigger reduction on means for scales of depression and anxiety. • After the intervention, more participants in the Intervention Group had no depression or anxiety symptoms than in the Control Group, but no statistical differences were found (*p* > 0.05). • Compared with participants with chronic disease, participants with no chronic disease had a statistically significantly larger reduction of total DASS-21 scale (mean difference on the DASS-21 scale = − 4.74, 95% CI: − 9.31 to − 0.17; *p* = 0.04). • The length of hospital stay was statistically significantly associated with a greater increase in anxiety in the Intervention Group (*p* = 0.005), whilst no statistically significant association was found in the Control Group (*p* = 0.29).
Liu, 2020 (RCT) [42]	China; Hospital.	Patients with confirmed COVID-19 (*N* = 51) (*n* = 25 intervention group; *n* = 26 control group): 56% male; mean age 50.41 years (SD: 13.04 years).	Progressive muscle relaxation. Performed for between 20–30 min per day over a period of five consecutive days. Follow-up: none.	Routine care.	**Outcome measures used:** State-Trait Anxiety Scale (STAI). • The t-test results showed that the average score of anxiety before intervention was not statistically significant [between the two groups] (*p* = 0.713), and the average score of anxiety after the intervention was statistically significant (Intervention group: Mean 44.96+/−(SD)12.68; Control group: Mean 57.15+/−(SD)9.24; *p* < 0.001).
Liu, 2021(a) (RCT) [43]	China; Hospital.	Patients with COVID-19 hospitalised infections (*N* = 140) (*n* = 70 intervention group; *n* = 70 control group); (59% female; 54% aged 36 years and over.	‘WeChat’ intervention group. Promoted during ward rounds and through daily broadcasts. Included daily broadcasts which provided knowledge about COVID-19 – including prevention, treatment, and recovery measures. Participants encouraged, through a WeChat app platform, to conduct self-introductions, make friends, share experiences, help each other build confidence, satisfy spiritual issues, and soothe stress. Follow-up: none.	Routine care.	**Outcome measures used:** State Anxiety Inventory (SAI). • Results showed that the average State Anxiety Inventory (SAI) score of the trial group was 38.5 ± 13.2, and it was 15.9% lower than the control group (45.8 ± 10.4) resulting in a statistically significant difference (*p* < 0.001). • Females, young, well-educated, and those without underlying diseases were more willing to involved in, and more vigorous to fulfil intervention activities and rehabilitation exercises; moreover, they found it easier to understand COVID-19 prevention methods and countermeasures.
Liu, 2021(b) (RCT) [44]	China; Hospital.	Patients with COVID-19 from five sites (*N* = 273) (*n* = 137 intervention group; *n* = 136 control group): 59% male; mean age intervention group: 43.76 years (SD: 14.31 years); mean age control group: 41.52 years (SD: 11.51 years).	Computerised Cognitive Behavioural Therapy (cCBT) A self-help intervention delivered through 10 min of self-directed individual therapy per day for 1 week at each trial centre. The cCBT intervention was installed on an iPad only available to research therapists. Therapists first show participants how to use the system before the participants can use the self-help intervention. Follow-up: one-month post-intervention.	Treatment as usual.	**Outcome measures used:** Hamilton Depression Rating Scale-17 (HAMD); Hamilton Anxiety Scale (HAMA), Self-Rating Depression Scale, Self-Rating Anxiety Scale. A mixed-effects repeated measures model revealed statistically significant improvement in depression (*p* < 0.001), anxiety (*p* < 0.001), during the post-intervention and follow-up periods in the intervention group compared to the control group.
Malboeuf-Hurtubise, 2021 (Cluster RCT) [45]	Canada; Schools.	Schoolchildren (*N* = 22): (*n* = 14 intervention group; *n* = 8 control group): mean age: 11.3 years; 50% female.	Emotion-based directed drawing intervention. The intervention was group-based, delivered online and remotely. Duration: 5 weeks (1 session per week with each session lasting approximately 45 mins). Content involved: story of a virus (Comic strip); drawing how you feel; drawing viruses with funny names; drawing what you are afraid of and putting it in a bottle and throwing this in the bin; drawing what makes you anxious and where you feel it in your body; drawing your COVID-19 cure; and forecasting how your heart feels. Follow-up: none reported.	Mandala drawing intervention. Group-based, delivered online and remotely.	**Outcome measure used:** Behaviour Assessment Scale for Children-3rd edition (BASC 111) • No statistically significant impact on levels of anxiety or depression in either the intervention or control group as measured by ANCOVA (*p* = 0.26 for anxiety; *p* = 0.68 for depression). • For anxiety: Intervention group had means (SD) pre-test: 3.71(1.48) and post-test: 3.5(1.70); Control group had means (SD) pre-test: 3.25(2.05) and post-test: 2.87(0.83). • For depression Intervention group had means (SD) pre-test: 2.46(1.71) and post-test: 2.07(1.49); Control group had means (SD) pre-test: 2.62(1.84) and post-test: 2.62(1.50).
Ng, 2006 (RCT) [46]	Hong Kong; Community.	Community Rehabilitation Network for participants with chronic disease (*N* = 51) (*n* = 25 intervention group; *n* = 26 control group): 74.5% female; mean age of intervention group: 53.9 years.	Strength-Focused and Meaning-Oriented Approach for Resilience and Transformation (SMART) debriefing intervention for people exposed to SARS. One-day psycho-educational intervention. Follow-up: one-month post-intervention.	No intervention	**Outcome measure used**: Brief Symptom Inventory (BSI) Paired t-tests were conducted for changes between baseline (T_0_) and immediately at the end of the session (T_1_) only for the intervention group: ▪ The Depression score dropped significantly (*p* < 0.01). ▪ The Anxiety, Somatization and Hostility scores did not. Repeated-measure ANOVA was conducted for changes between T_0_ and 1 month after the intervention (T_2_), comparing the intervention group (*N* = 21 valid responses) and the control group (*N* = 13 valid responses): ▪ Group effects were found in Personal-Positive (*p* < 0.01) and Social-Negative scores (*p* < 0.05); ▪ Depression was the only subscale in BSI which had statistically significant group effect (*p* < 0.05).
Ozlu, 2021 (RCT) [47]	Turkey; Hospital.	Patients receiving treatment in hospital for COVID-19 (*N* = 73 – data available for *N* = 67) (*n* = 33 intervention group; *n* = 34 control group): 44.77% female.	Progressive muscle relaxation exercises. Compact Disk (CD) provided to the intervention group (*N* = 33) and exercises performed twice a day for 5 days over a period of 3 months. Exercises took approximately 20-30 min to perform. Follow-up: none reported.	Routine care	**Outcome measures used:** State-Trait Anxiety Inventory (STAI) (Turkish Version). • No statistically significant differences were found between the state and trait anxiety levels of the groups, which were determined to be homogeneous (*p* > 0.05). • The experimental group’s mean ± SD post-test score on the State-trait Anxiety Inventory Scale (SAS) was 44.67 ± 5.41, and the control group’s mean ± SD post-test score on the SAS was 61.29 ± 7.95. • A statistically significant difference was found between their mean post-test SAS scores. The mean post-test SAS score of the control group was higher than that of the experimental group (*p* < 0.05). • A statistically significant difference was found between the experimental group’s mean pre-test and post-test SAS scores. • No significant differences were found between the mean pre-test and post-test SAS scores of the control group.
Parizad, 2021 (RCT) [48]	Iran; Community.	Patients with COVID-19 (*N* = 110) (*n* = 55 intervention group; *n* = 55 control group): 54.4% male in the intervention group; Mean age intervention group: 43.14 years (SD: 12.22 years).	Guided imagery under a psychiatrist’s supervision. Ten sessions for five consecutive days, twice a day for an hour and a half. Delivered by audio track via headphones, administered by a nurse. Instructional guided imagery tracks lasted approximately 25 mins. Five different audio tracks. During each session, the patient closed their eyes, took deep breaths, and relaxed their muscles. Then they moved towards relevant imagery using mind and imagination. Follow-up: none reported.	Routine care	**Outcome measure used**: Spielberger State-Trait Anxiety Inventory (STAI). • The results of the paired-samples t-test revealed that the mean scores of the state (*p* < 0.001) and the trait anxiety (*p* < 0.001) statistically significantly differed in the intervention group (pre- vs post-intervention). • The mean scores of the state (*p* = 0.214) and the trait anxiety (*p* = 0.629) did not have a statistically significant difference in the control group (pre- vs post-intervention). • The difference in the mean score of the state (Cohen’s d = 1.10) and trait anxiety (Cohen’s d = 1.07) in the guided imagery group after the intervention compared to before the intervention was large. • The difference in the mean score of the state (Cohen’s d = 0.16) and trait anxiety (Cohen’s d = 0.06) in the control group was small.
Perri, 2021 (RCT) [49]	Italy; Hospital.	Healthcare professionals requiring psychological support to manage ongoing trauma associated with work on COVID-19 hospital wards (*N* = 38) (*n* = 19 intervention group; *n* = 19 control group): 71.1% female.	Eye Movement Desensitization and Reprocessing (EMDR). Dispensed online by 8 experienced psychotherapists. Seven-sessions therapy: two sessions per week for a duration of approximately 3 weeks. Follow-up: 1 month.	Comparison between EMDR and Trauma-focused cognitive-behavioural therapy (TF-CBT). Dispensed online by six experienced psychotherapists. Seven-sessions therapy: two sessions per week for a duration of approximately 3 weeks.	**Outcome measures used:** Post Traumatic Syndrome Disorder (PTSD) Checklist for DSM-V (PCL-5) – *a self-report checklist to assess the presence and severity of PTSD symptoms*; State Trait Anxiety Inventory (STAI-Y1); Beck Depression Inventory (BDI-II) • No intervention was found to be superior to the other. The RM-ANOVAs effect of therapy and therapy time interaction did not reach statistical significance (all *p*-values > 0.05). • State anxiety decreased by approximately 30% in both intervention groups after the seven-session treatment. • Traumatic and depressive symptoms reduced by approximately 55% after the seven-sessions in both interventions. • RM-ANOVAs yielded a significant main effect of time for the PCL-5 (*p* < 0.0001), STAI Y-1 (*p* < 0.0001, 2p = 0.53) and BDI-II scores (*p* < 0.0001). • Post-hoc comparisons revealed similar values of EMDR and TF-CBT for all the considered measures, and a significant score decrease from pre- to post-treatment, and from pre-treatment to follow-up for both groups (all Bonferroni-corrected *p*-values< 0.0001). • Results were confirmed at a one-month follow-up.
Procaccia, 2021 (RCT) [50]	Italy; Hospital.	Healthcare workers (caring for patients with COVID-19) from two hospitals (*N* = 55) (*n* = 30 intervention group; *n* = 25 control group):54.4% nurses; 27.27% physicians; 18.18% allied healthcare workers; 74.54% female.	Expressive Writing (EW) Intervention. Participants asked to write for three consecutive days at home for 20mins each describing their thoughts, feelings, and moods. Duration of study: 3 months. Follow-up: none reported.	Neutral writing (NW) task. Participants asked to write at home for three consecutive days for 20 mins each time, but they were asked to describe their experiences objectively.	**Outcome measures used:** Beck Depression Inventory (BDI-II), Los Angeles Symptom Checklist *– a self-report instrument with 43 items which measures overall distress related to trauma exposure, overall PTSD symptomology severity and PTSD symptoms sub-scales*. • For the EW group - Statistically significant interaction effects were found for PTSD symptoms, depression symptoms, and Global Severity Index. • No effects for social support and resilience were found. • Plots showed that: (1) PTSD symptoms reduced significatively only in EW group (PTSD writing condition: *p* = 0.002) (2) depression symptoms reduced in EW group while it increased in NW group (depression writing condition: *p* = 0.02).
Shabahang, 2020 (RCT) [51]	Iran; College.	College students (*N* = 150) (*n* = 75 intervention group; *n* = 75 control group): age range:18 to 32 years.	Cognitive Behavioural Therapy (CBT) (10 × 90 min sessions, 5-day week). Duration unclear. Delivered by two CBT experts. Follow-up: none.	Unclear.	**Outcome measures used:** Short Health Anxiety Inventory, adapted to coronavirus disease anxiety, Somatosensory Amplification Scale, and Beck Depression Inventory–Second Edition. • The cognitive-behavioural intervention was effective for health anxiety and depression of healthy individuals with high levels of coronavirus anxiety. - F statistic for health anxiety) was significant at 0.01 level. - F statistic for depression was significant at 0.01 level. - Small effect sizes were obtained for anxiety and depression.
Thombs, 2021 (RCT) [52]	Australia, Canada, France, Mexico, Spain, UK and USA; Community.	Patients with self-reported systemic sclerosis diagnosis (*N* = 172) (*n* = 86 intervention group, *n* = 86 control group): mean age: 55 years (SD: 11 years); 94% female; 79% identified as White.	Scleroderma Patient centered Intervention Network COVID-19 Home-isolation Activities Together (SPIN-CHAT) (10 weeks: 4 weeks intervention comprising 3 × 90 min sessions per week, and 6 weeks follow-up). Video-conferencing group intervention providing education and practice with mental health coping strategies and social support to reduce isolation. Follow-up: 6 weeks post-intervention.	Waiting list participants received reminders to complete trial measures only. (They received the SPIN-CHAT intervention following the six-week post-intervention outcome assessment).	**Outcome measures used:** Patient-Reported Outcomes Measurement Information System (PROMIS). The primary outcome analysed in the intention-to treat population was anxiety symptoms (PROMIS Anxiety 4a version 1.0) immediately post-intervention. • The intervention did not significantly reduce anxiety symptoms post-intervention (score difference: − 1.57 points; 95% CI: − 3.59 to 0.45; standardised mean difference [SMD]: − 0.22 points) but reduced symptoms 6 weeks later (score difference: − 2.36 points; 95% CI: − 4.56 to − 0.16; SMD − 0.31 points). • Depression symptoms were significantly lower 6 weeks post-intervention (score difference: − 1.64 points, 95% CI: − 2·91 to − 0.37; SMD: − 0.31 points).
Vanden Bossche, 2021 (RCT) [53]	Belgium; Community (city of Ghent).	Patients living in Ghent and known by a family physician working in the same urban area (*N* = 135) (*n* = 67 intervention group; *n* = 68 control group): mean age: 60.4 years (range: 19 to 93 years); 62.2% female; 32.6% migrants.	Community Health Worker (CHW) intervention: *N* = 50 CHWs matched with *N* = 67 pairs of patients. 8 weeks of tailored psychosocial support for the intervention group. Follow-up: none after the 8 weeks (end of intervention).	Usual care.	**Outcome measures used:** Patient-Reported Outcomes Measurement Information System (PROMIS). • For anxiety and fear of COVID-19, independent-samples t-test results revealed a statistically significant difference (*p* = 0.049 and *p* = 0.017, respectively), despite random attribution to intervention and control group. The patients of the intervention group had higher scores (meaning higher levels of anxiety and fear of COVID-19) than the control group patients at baseline. • The 95% confidence intervals fell entirely between the margins of meaningful change of [5, + 5%], except for anxiety where the mean decrease in anxiety in the control group might be larger than in the intervention group. • For anxiety: baseline-adjusted mean difference between groups post-intervention: 2.32 (95% CI: − 1.89 to 6.52; *p* = 0.278).
Vukcevic Markovic, 2020 (RCT) [54]	Most probably Serbia, but not clearly specified; Community.	General population (*N* = 135) (*n* = 67 intervention group; *n* = 68 control group): 70% female in the control group; 77% female in the intervention group; mean age control group: 32.67 years; mean age intervention group: 31.79 years.	Expressive writing (EW) intervention. Five EW sessions, each lasting 20mins, set 3 days apart, over a 2 weeks period. In EW the participant is asked to disclose their deepest thoughts and feelings surrounding a stressful life event. The notion is that EW can decrease negative feelings, and improve physical and mental health, by engaging in deep and meaningful writing about a traumatic or difficult event. Follow-up: 1 month.	Usual care.	**Outcome measures used:** Depression Anxiety Stress Scale (DASS); WHO well-being index; Manchester Short Assessment of Quality of Life. • A main effect of group on post-test Depression and Anxiety, after controlling for baseline scores, was not found: *p* = 0.082, pη2 = 0.030 and *p* = 0.735, pη2 = 0.001, respectively. • The study found no evidence that five sessions of remote EW generate benefits in lowering depression, anxiety, and stress, and increasing overall well-being. • On the contrary, the results showed that engaging in EW during the pandemic elevates the stress level of participants from the intervention group. The same results were obtained when controlling for the baseline results.
Wahlund, 2021 (RCT) [55]	Sweden; Community.	Adults in Swedish population reporting daily uncontrollable worry about COVID-19 and its possible consequences (*N* = 670) (*n* = 335 intervention group; *n* = 335 control group): 42% with previous psychiatric diagnosis; 4% had confirmed COVID-19 diagnosis; 81.64% female.	Brief self-guided online psychological intervention. Three-week duration completely self-guided via encrypted website and organised into five brief modules. Follow-up: 1 month.	Waiting list of equal duration. Participants randomised to control group were crossed over to the intervention after the controlled study period (week 3).	**Outcome measure used:** COVID-19 adapted version of the Generalised Anxiety Disorder 7-item scale. • The primary intention-to-treat mixed effects regression model showed that both groups improved significantly over time (ß = 0.74–1.89; Z = 9.36–19.84; *p* < 0.001) but the intervention group had a larger reduction in COVID-19-related worry than the control group (ß = 1.14; Z = 9.27; *p* < 0.001). • The intervention was associated with significant improvements including depressive symptoms: GAD-7 from post-treatment to the 1-month follow-up (ß = 1.78; Z = 8.06; *p* < 0.001).
Zheng, 2021 (Cluster RCT) [56]	China; Schools (Grade 7) in the Duanzhou district of Zhaoqing City, Guangdong Province, Southern China.	Children. (*N* = 954) (*n* = 485 intervention group; *n* = 469 control group); in *n* = 6 schools: mean age: 13.5 years (SD: 0.5 years): 52.3% male.	Digital behaviour change intervention which included: health education information promoting exercise and ocular relaxation, access to digital behaviour change intervention, with live streaming and peer sharing of promoted activities. Duration and follow-up unclear – presumed to be 2 weeks (the only follow-up timepoint).	Health education information only.	**Outcome measures used:** Self-reported Spence Children’s Anxiety Scale (SCAS) and a parent questionnaire. The assigned interventions were completed by 896 children (intervention: *N* = 467, 96.3%; control: *N* = 429, 91.5%). • The 2-week change in square-root–transformed self-reported anxiety score was greater in the intervention (− 0.23;, 95% CI: − 0.27 to − 0.20) vs control group (0.12; 95% CI: 0.09 to 0.16); unadjusted difference: − 0.36, 95% CI: − 0.63 to − 0.08; *p* = 0.02). • In linear regression models, randomisation to receive the peer-to-peer live-streaming intervention was associated with a statistically significant reduction in self-reported anxiety compared to the controls (ß = − 0.36; 95% CI: − 0.63 to − 0.08; *p* = 0.02), after adjusting for sex and household income.

*Abbreviations*: *ANCOVA* Analysis of Covariance, BASC 111 - Behaviour Assessment Scale for Children-3rd edition, *BDI-II* Beck Depression Inventory scores, *BSI* Brief Symptom Inventory, *CBT* Cognitive Behavioural Therapy, *cCBT* computerised Cognitive Behavioural Therapy, *CD* Compact Disk, *CI* Confidence Interval, *CW* Community Health Worker, *DASS12* Chinese Version of the Depression Anxiety and Stress Scale-21, *EFT* Emotional Freedom Techniques, *EMDR* Eye Movement Desensitization and Reprocessing, *EW* Expressive Writing intervention, *HADS-A* Hospital Anxiety and Depression Scale – Anxiety, *HADS-D* Hospital Anxiety and Depression Scale – Depression, *HAMD and HAMA* Hamilton Depression Rating Scale-17 (HAMD); Hamilton Anxiety Scale (HAMA), *N* Number, *NW* Neutral Writing task, *PANAS* Positive and Negative Affect Schedule, *PCL5* tool used to quantify and monitor symptoms over time, to screen individuals and assist in making a provision diagnosis of PTSD, *PROMIS* Patient-Reported Outcomes Measurement Information System, *PTSD* Post Traumatic Shock Disorder, *RCT* Randomised Controlled Trial, *RM-ANOVAs* Repeated Measures – Analysis of Variance, *SAI* State Anxiety Inventory, *SAS* State-trait Anxiety Inventory Scale, *SD* Standard Deviation, *SMD* Standardised Mean Difference, *SMART* Strength-Focused and Meaning-Oriented Approach for Resilience and Transformation, *SPIN-CHAT* Scleroderma Patient centered Intervention Network COVID-19 Home-isolation Activities Together, *STAI-Y* State-Trait Anxiety Inventory, *STICA* State-Trait Inventory of Cognitive and Somatic Anxiety, *TF-CBT* Trauma-focused Cognitive-Behavioural Therapy, *WEMWBS* The Warwick-Edinburgh Mental Wellbeing Scale, *WHO-QOL* Quality of Life Assessment developed by the World Health Organisation

### Quality assessment

Most of the included studies were assessed as being of high risk of bias (*n* = 12/22), or of ‘some concern’ (*n* = 8/22). Two studies were assessed as being of low risk of bias [54, 55]. Studies that were considered as high risk or of ‘some concern’ showed shortcomings due to either their randomisation process, deviations from their intended interventions, missing outcome data, their measurement of outcomes, or selective reporting. Table 3 provides an overall summary of the individual risk of bias assessments for each of the included studies.

**Table 3:** Risk of bias (RoB2) assessments for studies included in the systematic review of the effectiveness of psychological support interventions for those exposed to mass outbreaks

Study	Domain 1. RoB from randomization process	Domain 2: RoB due to deviations from intended interventions (effect of assignment to intervention)	Domain 2: RoB due to deviations from intended interventions (effect of adhering to intervention)	Domain 3: Missing outcome data	Domain 4: RoB in measurement of outcomes	Domain 5: RoB in selection of reported result	Overall risk of bias judgement
Al Alawi, 2021 [35]	**+**	**?**	**+**	**+**	**?**	**+**	**?**
Carbone, 2021 [36]	**+**	**+**	**+**	**+**	**?**	**?**	**?**
Dincer, 2021 [37]	**?**	**?**	**+**	**+**	**+**	**+**	**?**
Fiol-DeRoque, 2021 [38]	**+**	**?**	**?**	**+**	**+**	**+**	**?**
Gharaati Sotoudeh, 2020 [39]	**?**	**?**	**–**	**–**	**?**	**+**	**_**
Kong, 2020 [40]	**+**	**?**	**–**	**+**	**+**	**+**	**?**
Li, 2020 [41]	**+**	**?**	**?**	**+**	**+**	**+**	**?**
Liu, 2020 [42]	**+**	**?**	**+**	**+**	**?**	**?**	**–**
Liu Y, 2021(a) [43]	**?**	**?**	**+**	**+**	**?**	**?**	**–**
Liu Z, 2021(b) [44]	**?**	**?**	**+**	**+**	**?**	**?**	**–**
Malboeuf-Hurtubise, 2021 [45]	**_**	**?**	**?**	**?**	**?**	**?**	–
Ng, 2006 [46]	**+**	**?**	**?**	**–**	**–**	**?**	–
Ozlu, 2021 [47]	**+**	**?**	**+**	**+**	**?**	**?**	**–**
Parizad, 2021 [48]	**+**	**?**	**+**	**+**	**?**	**?**	**–**
Perri, 2021 [49]	**?**	**?**	**+**	**?**	**?**	**?**	**–**
Procaccia, 2021 [50]	**?**	**?**	**+**	**?**	**?**	**?**	**–**
Shabahang, 2020 [51]	**+**	**+**	**+**	**+**	**?**	**?**	**?**
Thombs, 2021 [52]	**+**	**+**	**?**	**?**	**?**	**+**	**–**
Vanden, 2021 [53]	**+**	**+**	**+**	**+**	**?**	**+**	**?**
Vukcevic, 2020 [54]	**+**	**+**	**+**	**+**	**+**	**+**	**+**
Wahlund, 2021 [55]	**+**	**+**	**+**	**+**	**+**	**+**	**+**
Zheng, 2021 [56]	**?**	**?**	**?**	**+**	**+**	**+**	**–**

Key:

+ Low risk of bias

- High risk of bias

? Some concerns

### Year and location of studies

Most of the studies included in the review were published in 2020 or 2021 (*n* = 21). One study was published in 2006 [46]. Studies were conducted in several different countries: Belgium (*n* = 1) [53], Canada (*n* = 1) [45], China (*n* = 6) [40–44, 56], Hong Kong (*n* = 1) [46], Iran (*n* = 3) [39, 48, 51], Italy (*n* = 3) [36, 49, 50], Oman (*n* = 1) [35], Serbia (*n* = 1) [54], Spain (*n* = 1) [38], Sweden (*n* = 1) [55], and Turkey (*n* = 2) [37, 47]. One study [52] involved participants from seven different countries (Australia, Canada, France, Mexico, Spain, UK and USA). Nine of these studies’ countries were high-income countries (HICs) [35, 36, 38, 45, 46, 49, 50, 53, 55] and 12 were upper-middle-income countries (UMICs) [37, 39–44, 47, 48, 51, 54, 56]. One study was conducted in countries from both HIC and UMIC [52]. No published studies were identified from either in low-income-countries (LICs) or lower-middle-income countries (LMICs).

### Participant characteristics

#### Patients

Eleven studies involved participants who were patients, of which: seven involved patients with COVID-19 [39, 40, 42–44, 47, 48]; three with pre-existing chronic diseases exposed to COVID-19 and / or with a diagnosis of COVID-19 [41, 52, 53]; and one involved adult patients with chronic diseases exposed to SARS [46].

#### General population

Seven studies involved participants from general populations exposed to COVID-19 [35, 36, 45, 51, 54–56]. Two of these seven studies involved schoolchildren [45, 56] and one involved college students [51].

#### Healthcare workers

Four studies focused on healthcare workers caring for patients with COVID-19, specifically nurses and / or other hospital staff [37, 38, 49, 50]. No studies included social care workers. Only one study included staff from primary care settings [38].

#### Sample size

Participant numbers ranged from 22 to 954 (average number of participants: 173). The total number of participants included in the review was 3814.

#### Participant age and target population

Seventeen studies provided detailed age-related data [35–39, 41, 42, 44–48, 52–56]. Participants’ mean ages ranged from 11.3 years to 60.4 years. The average age of all 17 studies’ participants was 39.58 years. All studies provided gender-related data except for one study [51]. Fifteen of the 21 studies providing data involved more female participants than male participants (range: 51 to 94% female participants) [35–41, 43, 46, 49, 50, 52–56].

### Settings

Eleven studies were conducted in community-based settings [35, 36, 45, 46, 48, 51–56] and ten were conducted in hospitals [37, 39–44, 47, 49, 50]. One study included both hospital and community-based settings [38]. Only one study included primary care and long-term care facility settings [38].

### Interventions

All studies investigated psychological mental health (support) interventions for participants exposed to COVID-19 and / or with a diagnosis of COVID-19 apart from one, which examined interventions for adult patients with chronic medical conditions exposed to SARS [46].

#### Interventions for patients

Ten studies examined specific interventions for patients [39–44, 47, 48, 52, 53]. These interventions included: a brief crisis mental health intervention package [39]; a psychological behavioural intervention [40]; Cognitive Behaviour Therapy [41]; progressive muscle relaxation exercises [42, 47]; a group intervention [43]; computerised Cognitive Behavioural Therapy [44]; guided imagery under a psychiatrist’s supervision [48]; a tailored intervention and group app ‘SPIN-CHAT’ for patients with a chronic medical condition [52]; and a Community Health Worker intervention [53]. One study explored a culturally specific debriefing intervention for patients with chronic disease following a SARS outbreak [46].

#### Interventions for the general population

Seven studies investigated interventions for the general population [35, 36, 45, 51, 54–56]. These included: an internet-based therapist-guided online therapy focusing on symptoms of anxiety and depression in adults in the general population exposed to COVID-19 [35]; an online counselling session focusing on reducing clinical symptoms and increasing wellbeing amongst adults during the first COVID-19 lockdown in Italy [36]; an emotion-based directed drawing intervention for schoolchildren [45]; Cognitive Behavioural Therapy sessions for college students [51]; an Expressive Writing intervention for adults [54]; a brief self-guided online psychological intervention for adults reporting uncontrollable worry about COVID-19 [55]; and a digital behaviour change intervention aimed at reducing anxiety in schoolchildren [56].

#### Interventions for healthcare workers

Four studies explored interventions for healthcare workers exposed to COVID-19 [37, 38, 49, 50]. These interventions included: a brief (one online session lasting 20 min) form of an Emotional Freedom Techniques aimed at the prevention of stress and anxiety in nurses involved in the treatment of COVID-19 patients [37]; a psychoeducational mindfulness-based mHealth intervention for healthcare workers (nurses, physicians, and nursing assistants) working in either a hospital-based setting, primary care or homecare setting during the COVID-19 pandemic [38]; an Eye Movement Desensitization and Reprocessing (EMDR) intervention for healthcare workers working on COVID-19 hospital wards [49]; and an Expressive Writing intervention for healthcare workers (nurses, physicians and allied healthcare workers) caring for COVID-19 patients in hospital [50].

Twelve interventions were delivered remotely either online via the internet, by mobile phone apps, audio-recordings or by video-conferencing facilities [35, 36, 38, 43–45, 48, 49, 52, 54–56]. One intervention involved the use of an audio-recording played under supervision [47], and one intervention involved a writing task conducted alone at home [50]. The remainder were delivered in person (face-to-face).

Eight studies included a 4-6 weeks post-intervention follow-up period [35, 39, 44, 46, 49, 52, 54, 55]. Two studies included a 24-h to 10 days post-intervention follow-up [38, 40]. Six studies had no post-intervention follow-up [36, 37, 42, 43, 51, 53]. The follow-up for the remaining six studies was unclear [41, 45, 47, 48, 50, 56].

### Comparators

All studies included comparators which were either: no intervention [37, 46]; routine (usual) care [39–44, 47, 48, 53, 54]; waiting lists [36, 52, 55]; a newsletter or brief written (health related) information (only) [35, 38]; a different psychological support intervention [45, 49]; or a neutral task e.g. neutral writing task instead of an expressive writing intervention [50, 56]. The comparator in one study was unclear [51].

### Meta-analysis

**Fig. 2:**
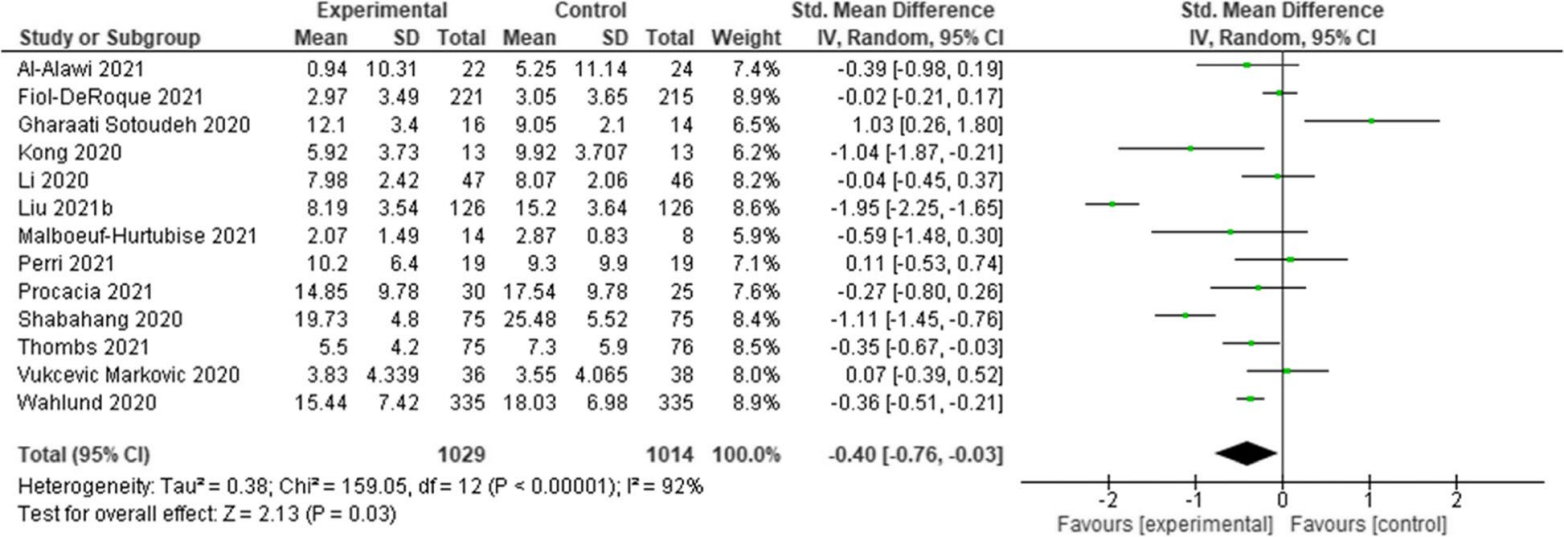
Comparative effectiveness of psychological intervention on measures of depression

**Fig. 3:**
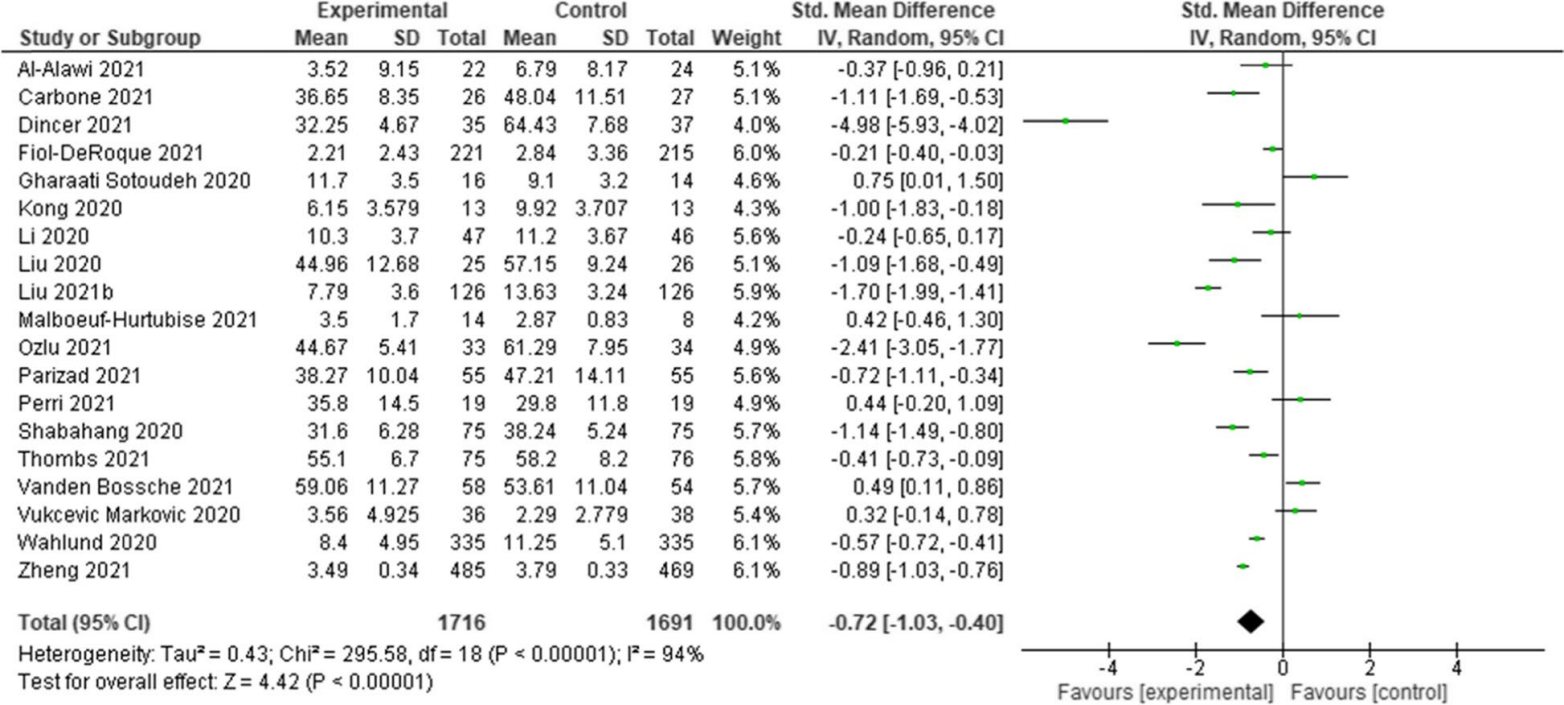
Comparative effectiveness of psychological intervention on measures of anxiety

**Fig. 4:**
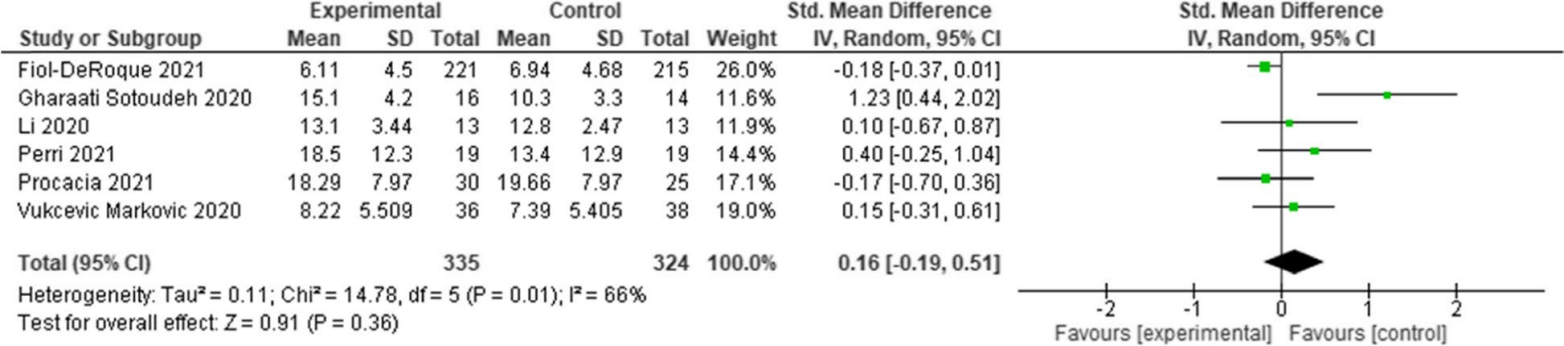
Comparative effectiveness of psychological intervention on measures of stress

Meta-analyses, involving all but two of the included studies [43, 46], showed that psychological interventions had a statistically significant benefit in managing depression (Standardised Mean Difference [SMD]: -0.40; 95% Confidence Interval [CI]: − 0.76 to − 0.03) (Fig. 2) and anxiety (SMD: -0.72; 95% CI: − 1.03 to − 0.40) (Fig. 3). The effect on stress was equivocal (SMD: 0.16; 95% CI: − 0.19 to 0.51) (Fig. 4). All analyses were affected by substantial or considerable heterogeneity (I^2^ 92% (depression); I^2^ 94% (anxiety); and I^2^ 66% (stress)). The two studies excluded from the meta-analysis included culturally specific psychological interventions for patients with mild-COVID-19 [43], and patients with chronic diseases following a SARS outbreak [46]. These studies reported benefits in managing anxiety [43]; and depression [46].

### Sensitivity and sub-group analyses

Sensitivity analyses were also conducted, removing studies which appeared to skew the findings [37, 39] for depression, anxiety and stress (Additional file 3). Although heterogeneity was markedly reduced for the meta-analysis of stress outcome, it had limited effect on both the heterogeneity associated with the analyses of depression and anxiety and the pooled effect measures for all outcomes.

Sub-group post-hoc analyses were conducted after looking at the review’s data to investigate the influence of different participants, the setting of the interventions and the RCTs’ risk of bias (Additional file 3). Heterogeneity continued to affect the different sub-groups, with limited variation in the outcomes from the original meta-analyses. It was evident from the sub-group analysis based on risk of bias, that studies of depression and anxiety that were at a higher risk of bias tended to report greater benefit from the intervention. However, as most of the meta-analyses were affected by considerable heterogeneity, they should be interpreted cautiously.

## Discussion

This is the first systematic review which aimed to identify the types of psychological (mental health) interventions which have been used either in previous mass infectious disease outbreaks (similar to COVID-19) or during the COVID-19 pandemic to support the general population and healthcare workers, and to assess how effective they have been. The meta-analyses conducted suggest that different psychological support interventions have shown potential effectiveness to reduce levels of anxiety and depression in those exposed to mass infectious disease, but not for levels of stress. This finding supports other sources which argue that whilst individually-directed psychological interventions are associated with some reductions in mental health conditions such as depression and anxiety, there needs to be a more holistic approach which includes both personalised interventions and organisational-level and societal structural changes to decrease stressors associated with a mass outbreak [10–12]. For example, healthcare workers continuously working long hours in stressful and resource constrained settings [12] require organisational-level interventions to improve their working environments and thereby decrease associated stressors, yet there is limited evidence of preventative measures or organisational level interventions [10]. Also, prior to a mass outbreak, members of the general population may already have experienced an ongoing lack of mental health service provision, but this gap has been exacerbated during the COVID-19 pandemic [10–12]. Wider public mental health provision should, therefore, be included in national preparedness and emergency response plans for mass infectious disease outbreaks [10–12].

Many of the interventions identified by this review were delivered remotely e.g. online or by a mobile phone app rather than in person. Such resources may be helpful in helping in managing and coping with the mental health impacts arising from a mass outbreak. Establishing more online mental health services in hospitals and communities could be an opportunity to address the shortage of mental health care service provision for the general population and healthcare workers [12]. However, further studies are needed to explore their effectiveness and their acceptability to the recipients. Consideration is also needed for all those who may not have access to online digital resources and / or support to help them access these resources.

Our review found a lack of RCTs with representative study populations including: insufficient studies involving healthcare workers (*n* = 4/22); none involving social care workers; few involving children and young people (*n* = 2/22); few involving primary care staff (*n* = 1/22); a lack of studies involving male participants (*n* = 15/22 studies involved higher percentages of female participants); and no studies from LICs or LMICs.

Many of the interventions were brief, involving small sample sizes and with little or no longer-term follow-up: 12 studies had no follow up or they did not report on their follow-up. The longest follow-up period post-intervention was 6 weeks. Most of the studies were assessed as being of high risk of bias, which appeared to affect outcomes. Some of the brief interventions involved participants re-living past traumatic experiences. Brief single-session debriefing interventions that focus on re-living past experiences for those who have experienced a traumatic event are not recommended as they may increase their risk of depression and Post Traumatic Shock Disorder (PTSD) [20, 57].

An influenza pandemic of unprecedented scale was expected many years before the outbreak of COVID-19 [1–5]. The likely impact and the need for psychological interventions to support and build the resilience of those exposed were also known [11, 13]. Despite this, our review found a lack of published evidence exploring the effectiveness of psychological interventions for those exposed to such mass infectious disease outbreaks. Most of the evidence identified by the review was related to COVID-19 and published in the last 15 months. This suggests that lessons have not been learnt from previous mass infectious disease outbreaks. The focus of previous published research may have been concerned with a focus on the mass outbreak itself and not with its wider consequences on mental health outcomes; or it may have been concerned with exploring interventions for those exposed to man-made or natural disasters such as war or earthquakes; or it may have been concerned with only certain population groups such as healthcare workers (only) or children (only). We acknowledge that the current pandemic is rapidly evolving globally, as is the evidence base. We also acknowledge that organisations may already provide interventions for mass infectious disease outbreaks, but that the effectiveness of these interventions may have not been formally evaluated in a mass outbreak setting. For example, NHS England commissioned resilience hubs for healthcare staff in late 2020 [58]. The effectiveness and acceptability of these hubs will need evaluating. There may be interventions that, while used in other situations (for example war settings), might have been used during the COVID-19 pandemic without prior testing in mass outbreak situations. It is not known if the types of interventions from other settings are effective in response to a mass outbreak such as COVID-19. Furthermore, findings from some relevant and ongoing COVID-19 related mental health support intervention trials may not have been published in peer-reviewed journals at the time of writing. In addition, the burden of care in the COVID-19 pandemic included staff working in long-term care home facilities and primary care staff as well as hospital-based staff. Our review found a dearth of research involving interventions for these staff. The implementation and impact of interventions in different healthcare workers and in different population groups may differ. Factors such as acceptability, access, take-up and timing of interventions for different healthcare and population groups are important considerations in trials and evaluations of complex interventions. Therefore, there are opportunities for further research including research to investigate whether interventions used in other large-scale disasters are comparable when used in response to a pandemic such as COVID-19, and to explore whether any organisations already offer any psychological interventions and to formally evaluate these.

This review’s findings support the call for greater global action [59]. Psychological interventions are needed for all those affected by, and / or more vulnerable to, the mental health consequences of a mass outbreak including, for example:

- ❖ Children and young people experiencing anxiety and depression associated with the loss of freedom and opportunities for play, boredom, separation from friends, and school closures during lockdowns [28].
- ❖ Ethnic minorities: a survey of over 14,000 people from ethnic minorities revealed that existing inequalities in housing, employment, finances, and other issues have had a greater impact on their mental health than white people during the COVID-19 pandemic [27].
- ❖ Healthcare workers experiencing anxiety, depression, stress or PTSD as a consequence of caring for populations affected by a mass outbreak. Healthcare workers carry significant burdens during a mass outbreak, including a considerable impact on their psychological wellbeing. Therefore, supporting their mental health wellbeing should continue to be a priority [8–10, 19]. This should include preventative measures as well as timely access to effective and acceptable psychological interventions where needed.
- ❖ People with disabilities such as those with learning disabilities, and those with pre-existing physical and mental health conditions during periods of shielding or self-isolation. The COVID-19 pandemic has exposed pre-existing health inequalities for people with mental health and learning disabilities [60].
- ❖ People experiencing mental health problems as a consequence of domestic violence. Domestic violence has increased during lockdowns as there is no escape from abusers [61].

All those affected need access to safe, appropriate, targeted evidence-based psychological interventions to support them both in the immediate and longer-term.

### Limitations

Our review was limited to studies published in English and there may be other relevant studies published in other languages. We originally intended to include all study types in our review (as indicated within our published protocol). However, the evidence base has evolved rapidly, and we were able to identify new experimental controlled studies that theoretically provided more robust evidence. The following studies limitations impinge on their generalisability: small sample sizes; challenges regarding recruitment and retention of representative samples of participants (particularly for studies conducted during a pandemic); high risk of (or some concerns with) bias; and / or a lack of follow-up to estimate the longer-term effects of an intervention. Some studies relied solely on self-reporting measures and participants may have provided positive responses as they may have wanted to please the researchers and / or they may have possibly feared losing their jobs / or treatment if they gave negative responses (response bias). Only those interested in the research, who had the time, were accessible or were paid for their involvement may have participated. Some improvements in mental health may have been the result of other confounding factors.

## Conclusions

Despite benefits from psychological interventions in managing anxiety and depression for all those exposed to mass infectious disease outbreaks, the evidence is limited. The review highlights the need for further research including complex intervention trials, studies involving representative study populations, studies to investigate whether interventions used in other large-scale disasters are effective when used during a pandemic such as COVID-19, and to explore whether any organisations already offer any psychological interventions, and to formally evaluate these. Research is needed now to inform the development and implementation of effective psychological interventions for all those exposed to mass infectious disease outbreaks to ensure we are adequately prepared. Intervention development, piloting, evaluating, reporting and implementation should follow recommended guidance for complex interventions [62, 63].

## Supplementary Information

**Additional file 1.** Search Strategy.

**Additional file 2.** Screening Tool.

**Additional file 3.** Sensitivity and sub-group meta-analyses.

## Data Availability

All data generated / analysed in this study are included in this published article / supporting files.

## Abbreviations

CBT: Cognitive Behavioural Therapy
COVID-19: Coronavirus
DASS-21: Depression, Anxiety and Stress Scale
EMDR: Eye Movement Desensitization and Reprocessing
GAD-7: Generalized Anxiety Disorder scale.
H1N1: A type of influenza A virus
HIC: High-income-country
LIC: Low-income-country
LMIC: Lower-middle-income-country
MERS: Middle East Respiratory Syndrome
PHQ-9: Patient Health Questionnaire depression scale.
PTSD: Post-Traumatic Shock Disorder
RCT: Randomised Controlled Trial
RoB2: Risk of bias assessment
SARS: Severe Acute Respiratory Syndrome
SARS-CoV-2: A virus that can cause COVID-19, a mass infectious disease
SMART: ‘Strength-Focused and Meaning-Orientated Approach for Resilience and Transformation’
UMIC: Upper-middle-income-country
