# Supplementary material for: The effectiveness of psychological support interventions for those exposed to mass infectious disease outbreaks: a systematic review
Source: BMC Psychiatry. 2021 Nov 24;21:592. doi: 10.1186/s12888-021-03602-7 (PMC8610770; doi:10.1186/s12888-021-03602-7)
Supplement: Supplementary file 1 — Additional file 1. Search Strategy. [file 12888_2021_3602_MOESM1_ESM.docx]

**Additional File 1**

**Search Strategy**

| 1. exp Disasters/ |
| --- |
| 1. epidemics/ or pandemics/ |
| 1. (outbreak$1 or pandemic* or epidemic or epidemics or ebola or H1N1 or swine flu or SARS or severe acute respiratory syndrome or Middle East Respiratory Syndrome or MERS).tw. |
| 1. (humanitarian adj3 (aid or affair* or agenc* or assistance or catastrophe* or crisis or crises or disaster* or effort* or emergenc* or evacuation* or integration or reintegration or mission or organization* or organisation* or program* or relief or setting* or support* or task force or work*)).tw. |
| 1. (cataclysmic or catastroph* or devastation or disaster* or drought* or earthquake* or evacuation* or famine* or flood or floods or hurricane or cyclone* or landslide* or land slide* or mass casualt* or tsunami* or tidal wave* or volcano*).tw. |
| 1. or/1-5 |
| 1. exp Psychotherapy/ |
| 1. exp Mental Health Services/ |
| 1. Anxiety Disorders/th [Therapy] |
| 1. Depressive Disorder/th [Therapy] |
| 1. exp "Trauma and Stressor Related Disorders"/th [Therapy] |
| 1. (psycholog* adj5 (therap* or service* or intervention* or treatment*)).tw. |
| 1. ((cognitive or behavio?r* or family or narrative or person* or acceptance or compassion* or art or music or drama or dance or play or gestalt or talk* or aversive or implosive or exposure or relaxation or emotion* or mentali?ation) adj3 therap*).tw. |
| 1. behavio?r* activation.tw. |
| 1. (eye movement desensiti?ation and re?processing).tw. |
| 1. emdr.tw. |
| 1. psychotherap*.tw. |
| 1. counselling.tw. |
| 1. bibliotherap*.tw. |
| 1. psychoanalytic.tw. |
| 1. biofeedback.tw. |
| 1. cognitive remediation.tw. |
| 1. cbt.tw. |
| 1. mindfulness.tw. |
| 1. or/7-22 |
| 1. meta-analysis.pt. |
| 1. meta-analysis/ or systematic review/ or meta-analysis as topic/ or "meta analysis (topic)"/ or "systematic review (topic)"/ or exp technology assessment, biomedical/ |
| 1. ((systematic* adj3 (review* or overview*)) or (methodologic* adj3 (review* or overview*))).ti,ab,kf,kw. |
| 1. ((quantitative adj3 (review* or overview* or synthes*)) or (research adj3 (integrati* or overview*))).ti,ab,kf,kw. |
| 1. ((integrative adj3 (review* or overview*)) or (collaborative adj3 (review* or overview*)) or (pool* adj3 analy*)).ti,ab,kf,kw. |
| 1. (data synthes* or data extraction* or data abstraction*).ti,ab,kf,kw. |
| 1. (handsearch* or hand search*).ti,ab,kf,kw. |
| 1. (mantel haenszel or peto or der simonian or dersimonian or fixed effect* or latin square*).ti,ab,kf,kw. |
| 1. (met analy* or metanaly* or technology assessment* or HTA or HTAs or technology overview* or technology appraisal*).ti,ab,kf,kw. |
| 1. (meta regression* or metaregression*).ti,ab,kf,kw. |
| 1. (meta-analy* or metaanaly* or systematic review* or biomedical technology assessment* or bio-medical technology assessment*).mp,hw. |
| 1. (medline or cochrane or pubmed or medlars or embase or cinahl).ti,ab,hw. |
| 1. (cochrane or (health adj2 technology assessment) or evidence report).jw. |
| 1. (comparative adj3 (efficacy or effectiveness)).ti,ab,kf,kw. |
| 1. (outcomes research or relative effectiveness).ti,ab,kf,kw. |
| 1. ((indirect or indirect treatment or mixed-treatment) adj comparison*).ti,ab,kf,kw. |
| 1. or/27-42 |
| 1. exp Health Personnel/ |
| 1. (personnel or staff or worker* or volunteer* or employee* or professionals or nurse* or doctor* or physician* or therapist* or psychologist* or assistant* or practitioner* or carer* or caregiver* or firefighter* or firem?n or first responder* or paramedic*).mp. [mp=title, abstract, original title, name of substance word, subject heading word, floating sub-heading word, keyword heading word, organism supplementary concept word, protocol supplementary concept word, rare disease supplementary concept word, unique identifier, synonyms] |
| 1. 44 or 45 |
| 1. 6 and 25 and 43 and 46 |
