# Supplementary material for: The effectiveness of psychological support interventions for those exposed to mass infectious disease outbreaks: a systematic review
Source: BMC Psychiatry. 2021 Nov 24;21:592. doi: 10.1186/s12888-021-03602-7 (PMC8610770; doi:10.1186/s12888-021-03602-7)
Supplement: Supplementary file 2 — Additional file 2. Screening Tool. [file 12888_2021_3602_MOESM2_ESM.docx]

| **Screening tool**  **Systematic review: Effectiveness of psychological interventions for the general population and healthcare workers exposed to mass infectious disease outbreaks** | |
| --- | --- |
| **Primary research question:**   1. To identify what interventions (i.e. psychological support) have been used in similar situations to Covid-19 and how effective they have been (for the general population and healthcare workers) compared to other approaches. | |
| **Questions - key elements** | |
| **Population (P)** | **Included:**   - General population / patients / survivors / victims of mass outbreaks and their carers / families. - Healthcare workers involved in mass outbreaks (all those who come into contact with and/ or support those coming into contact with patients in delivering forms of care). - Families of healthcare workers involved in mass outbreaks. |
|  | **Excluded:**   - Armed forces - Emergency services e.g. police, fire and rescue - Charity workers / NGOs |
| **Interventions (I)** | **Included**:   - Psychological interventions (pre-mass outbreaks, during outbreaks, post- outbreaks) - Psychosocial interventions |
|  | **Excluded**: pharmacological interventions |
| **Settings** | **Included**:   - Any type of mass outbreak e.g. 1918 Flu, 2002 SARS, Ebola, Cholera. - Mental health disorders e.g. depression disorders, anxiety and mass-outbreak-led PTSD. - Any country. |
|  | **Excluded**:   - War and conflict. - Terrorism e.g. 9/11. - Physical, sexual and domestic violence, abuse, torture, genocide. - Chemical, biological, radiological, or nuclear events (CBRN). - Man-made or natural disasters e.g. air disasters, tsunami, volcano, earthquakes, flooding. - PTSD caused by generic trauma. - HIV/AIDS. |
| **Outcomes (O)** | **Included:**   - Measure of the prevention / treatment of psychological problems (in general population and front-line healthcare workers). |
| **Comparison (C)** | **Any** |
| **Study design** | **Included:**  RCTs. All years. |
|  | **Excluded**: editorials, commentaries, abstracts. |
| **Limits** | English language studies only. |
