# Supplementary material for: The effectiveness of psychological support interventions for those exposed to mass infectious disease outbreaks: a systematic review
Source: BMC Psychiatry. 2021 Nov 24;21:592. doi: 10.1186/s12888-021-03602-7 (PMC8610770; doi:10.1186/s12888-021-03602-7)

**Additional file 3 Sensitivity and sub-group meta-analyses**

**Figure 5: Sensitivity analysis excluding Dincer et al., 2021; and Gharaati Sotoudeh et al., 2020: Comparative effectiveness of psychological interventions on measures of depression**


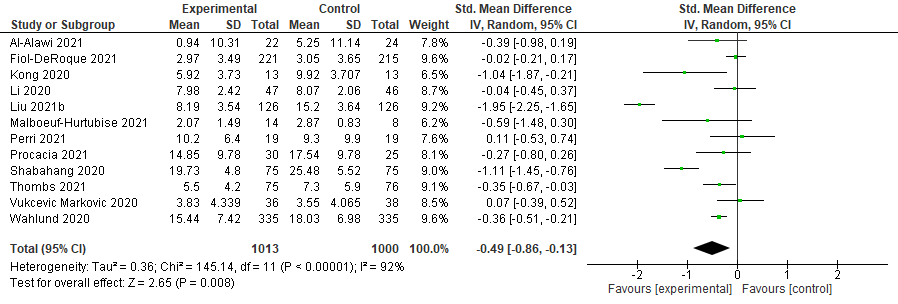


**Figure 6: Sensitivity analysis excluding Dincer et al., 2021; and Gharaati Sotoudeh et al., 2020: Comparative effectiveness of psychological interventions on measures of anxiety**


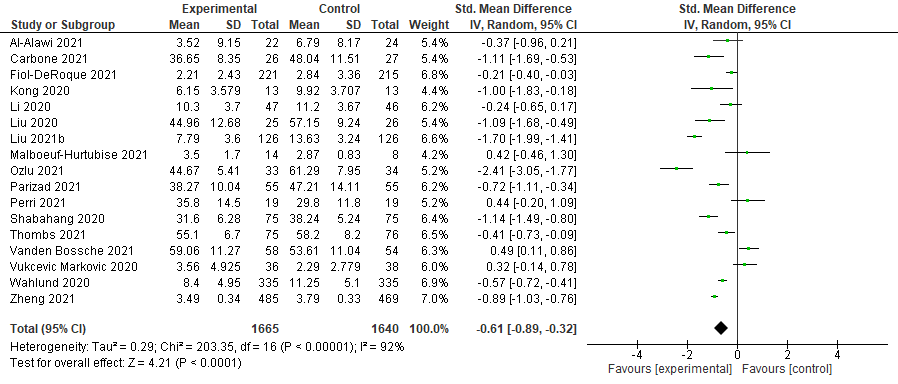


**Figure 7: Sensitivity analysis excluding Dincer et al., 2021; and Gharaati Sotoudeh et al., 2020: Comparative effectiveness of psychological interventions on measures of stress**


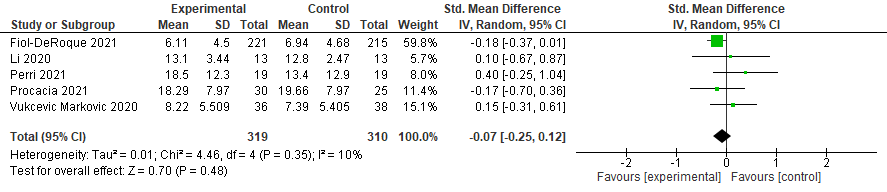


**Figure 8: Comparative effectiveness of psychological interventions on measures of depression by study risk of bias**


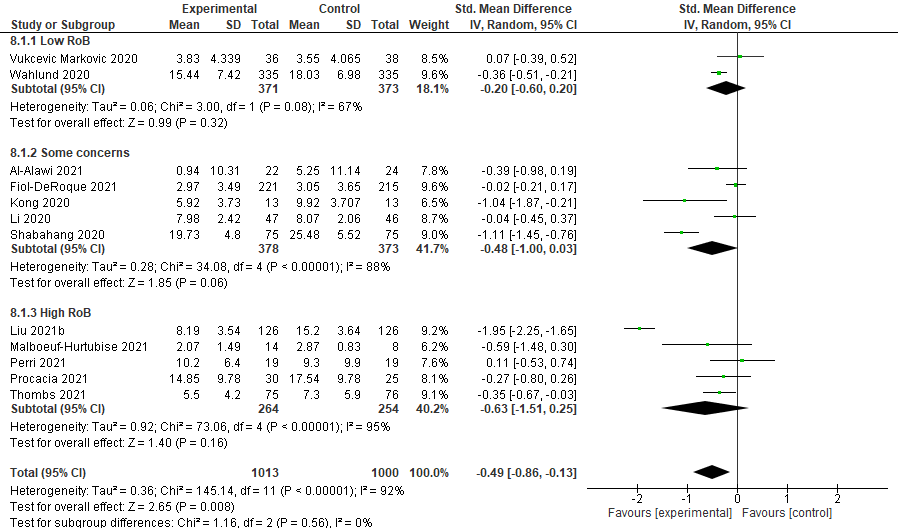


**Figure 9: Comparative effectiveness of psychological interventions on measures of anxiety by study risk of bias**


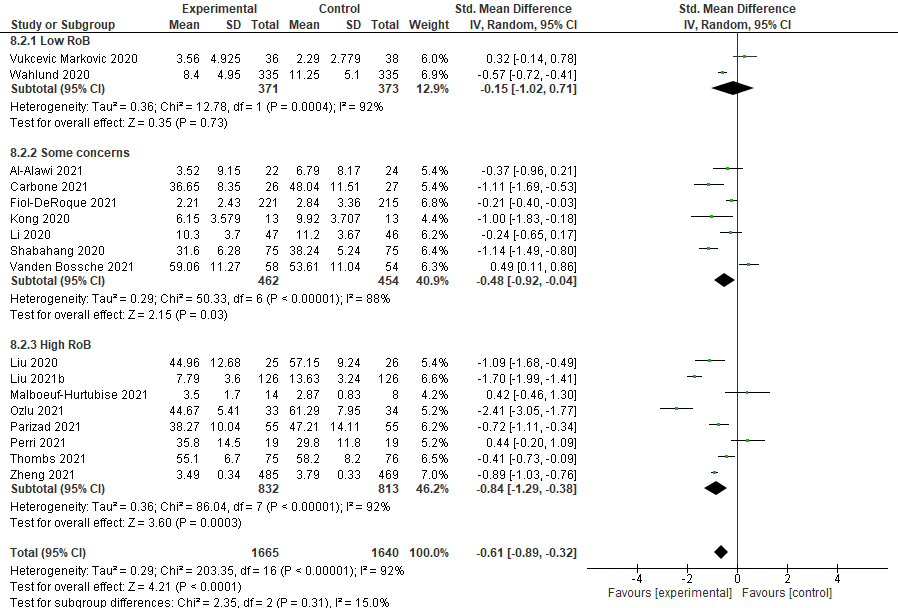


**Figure 10: Comparative effectiveness of psychological interventions on measures of stress by study risk of bias**


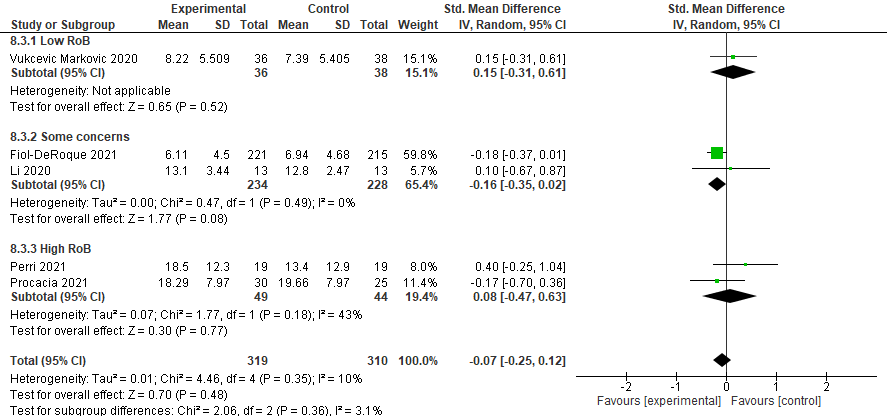

Supplement: Supplementary file 3 — Additional file 3. Sensitivity and sub-group meta-analyses. [file 12888_2021_3602_MOESM3_ESM.docx]
